# Revisiting the Immigrant Epidemiological Paradox: Findings from the American Panel of Life 2019

**DOI:** 10.3390/ijerph18094619

**Published:** 2021-04-27

**Authors:** Hans Oh, Jessica Goehring, Louis Jacob, Lee Smith

**Affiliations:** 1 Suzanne Dworak Peck School of Social Work, University of Southern California, 1149 Hill Street Suite #1422, Los Angeles, CA 90015, USA; jgoehrin@usc.edu; 2 Research and Development Unit, Parc Sanitari Sant Joan de Déu, CIBERSAM, Dr. Antoni Pujadas, 42, Sant Boi de Llobregat, 08830 Barcelona, Spain; louis.jacob.contacts@gmail.com; 3 Faculty of Medicine, University of Versailles Saint-Quentin-en-Yvelines, 78180 Montigny-le-Bretonneux, France; 4 The Cambridge Centre for Sport and Exercise Sciences, Anglia Ruskin University, Cambridge CB1 1PT, UK; lee.smith@aru.ac.uk

**Keywords:** immigrant, paradox, health advantage, health

## Abstract

Objective: Immigrants enjoy a health advantage over their US-born counterparts (termed the immigrant paradox), though the extent of this paradox may not extend to all health outcomes. Methods: We analyzed data from the RAND American Life Panel. Using multivariable logistic regression, we examined the associations between immigrant status and a wide range of health outcomes (e.g., cardiovascular diseases, mental health), adjusting for sociodemographic characteristics. Results: Being an immigrant was associated with lower odds of having any health condition, multimorbidity, and number of health conditions. When looking at specific conditions, however, immigrant status was only significantly associated with lower odds of depression, nerve problem causing numbness or pain, and obesity, but not other conditions. Conclusion: The immigrant paradox is evident when examining overall health, and specifically depression, nerve problems, and obesity.

## 1. Introduction

According to Pew Research Center [1], 40 million immigrants reside in the United States today (roughly 20% of the global migrant population), and this number is projected to grow to 78.2 million by 2065. This has considerable implications for population health and health care service systems. However, the literature has shown that an “immigrant paradox” exists such that immigrants exhibit a health advantage over their US-born counterparts despite facing more health risk factors (e.g., discrimination, lower socioeconomic status, barriers to healthcare). For example, studies have shown that immigrants have lower risk for mental health and substance use disorders [2,3] and mortality [4]. However, this paradox appears to only apply to certain conditions and not uniformly across all aspects of health and wellness [5,6]. Using data representative of the general adult population of the United States, we examined the association between immigrant status and a wide range of mental and physical health outcomes to determine the extent of the health advantage.

## 2. Methods

### 2.1. Sample

This study analyzed publicly available data from the RAND American Life Panel (ALP) [7], which is a US nationally representative probability-based panel consisting of approximately 6000 adults aged 18 and older. These panel members were recruited using probability sampling methods (e.g., address-based samples and telephone (random-digit dial) samples). To enhance representativeness, RAND provides computers and internet service to members who would not otherwise be able to participate. In 2019, RAND administered two surveys drawing respondents from the panel: the first was the ALP Omnibus Survey (*n* = 2555) conducted in February–April, which had a completion rate of 64.9%; and the second was the Health and Functional Capacity Survey (*n* = 2657) conducted April–June, which had a completion rate of 78.2%. All of the panel members who completed the ALP Omnibus Survey were included in the Health and Functional Capacity Survey, and so we merged the two datasets to yield a final analytic sample of *n* = 2554 for the current study. All panel members provided information on a standard set of sociodemographic characteristics in order to participate in the panel. Sampling weights were constructed to account for nonresponse and probability of selection using population distributions from the Current Population Survey Annual Social and Economic Supplement (provided by the U.S. Census Bureau). Data collection and survey protocols were approved by RAND’s Human Subjects Protection Committee, which serves as RAND’s Institutional Review Board.

### 2.2. Measures

#### 2.2.1. Immigrant Status (Independent)

Immigrant status was coded dichotomously to reflect foreign-born vs. US-born (reference).

#### 2.2.2. Health Conditions (Dependent)

Health conditions were self-reported (yes/no) and included 68 conditions, listed in Table S1. We created three outcomes, including any health condition, multimorbidity (at least two health conditions), and a count of health conditions.

#### 2.2.3. Sociodemographic Characteristics (Covariates)

Sociodemographic covariates included sex (male, female), age (continuous), and race/ethnicity (non-Hispanic White, non-Hispanic Black, Hispanic/Latinx, Other), education (less than high school, some high school but no diploma, high school graduate or equivalent, some college but no degree, professional school degree, Associate’s degree, Bachelor’s degree, Master’s degree, Doctoral degree), income (less than $25,000, $25,000–49,999, $50,0000–74,999, $75,000–99,999, $100,000–124,999, $125,000–199,999, $200,000 or more), and health insurance coverage (yes/no).

### 2.3. Analysis

We first examined the prevalence of health outcomes across immigrant status (Table 1). Using multivariable logistic regression, we examined the associations between immigrant status and any health outcome as well as multimorbidity (at least two health conditions) adjusting for sociodemographic characteristics. We then used negative binomial regression to examine the association between immigrant status and the count of conditions (Table 2). In a set of exploratory analyses, we examined the prevalence of each specific health condition across immigrant status (Supplemental Materials, Table S1). We ran bivariate logistic regression models examining the associations between immigrant status and each health outcome (Supplemental Materials, Table S2). We then used multivariable logistic regression to examine conditions that were statistically significant in bivariate models (Supplemental Materials, Table S3); significant associations are depicted in Table 3. We reported effects sizes as odds ratios with 95% confidence intervals (a = 0.05). We used STATA SE 15 (Stata Corp, College Station, TX, USA) to conduct all analyses.

## 3. Results

Approximately 71% of the sample had at least one health condition, and the prevalence was significantly higher among US-born respondents than among foreign-born respondents. Just over half of the sample had multimorbidity, which again, was more prevalent among US-born than among foreign-born. The average count of conditions was 2.49, and the average was significantly higher among US-born than among immigrants. Immigrants respondents were less likely to have insurance, had less education, were more likely to be a person of color (Latinx, Black, Other), and were younger.

Foreign-born respondents were significantly less likely to have any health condition and less likely to have multimorbidity when compared with their US-born counterparts (Table 2). Put differently, US-born respondents were 1.41 times as likely to have any health condition (aOR: 1.41; 95% CI: 1.06–1.89), and 1.32 times as likely to have multimorbidity (aOR: 1.32; 95% CI: 1.01–1.73). When examining the count of conditions, the incident rate for US-born respondents was 1.28 times the incident rate of foreign-born respondents.

**Table 2:** Associations between immigrant status and health conditions.

Immigrant Status	Any Condition (Logistic Regression) aOR (95% CI)		Multimorbidity (Logistic Regression) aOR (95% CI)		Number of Conditions (Negative Binomial Regression) IRR (95% CI)	
	I	II	I	II	I	II
Foreign-born	0.60 (0.47–0.78) *p* = 0.00	0.71 (0.53–0.94) *p* = 0.02	0.66 (0.52–0.84) *p* = 0.00	0.75 (0.58–0.99) *p* = 0.04	0.76 (0.65–0.90) *p* = 0.00	0.78 (0.67–0.92) *p* = 0.00
US-born	1.00	1.00	1.00	1.00	1.00	1.00
*N*	2555	2532	2555	2532	2555	2532

I. Bivariate. II. Adjusted for age, sex, education, household income, race/ethnicity, insurance status.

Descriptive statistics showing the prevalence of each health condition across immigrant status are presented in the supplemental materials (Table S1), as are simple bivariate associations using logistic regression (Table S2). The associations were largely nonsignificant, with the exception of arthritis, nerve problem causing numbness or pain, other heart or circulatory system disorder, depression, and obesity. However, after adjusting for sociodemographic characteristics (Table S3), only nerve problem causing numbness or pain, depression, and obesity were significantly lower for foreign-born respondents when compared with US-born respondents. In other words, being born in the US was associated with 1.81 times greater odds of nerve problem causing numbness or pain (aOR: 1.81; 95% CI: 1.08–3.02), nearly double the odds of depression (aOR: 1.99; 95% CI: 1.26–3.17), and 1.60 times greater odds of obesity (aOR: 1.60; 95% CI: 1.02–2.51) (Table 3).

**Table 3:** Associations between immigrant status and specific health conditions.

Immigrant Status	Nerve Sroblem Causing Numbness or Pain	Depression	Obesity
	aOR (95% CI)	aOR (95% CI)	aOR (95% CI)
Foreign-born	0.55 (0.33–0.93) *p* = 0.02	0.50 (0.32–0.80) *p* = 0.00	0.63 (0.40–0.98) *p* = 0.04
US-born	1.00	1.00	1.00

Adjusted for age, sex, education, household income, race/ethnicity, insurance status.

## 4. Discussion

Consistent with the immigrant paradox, we found that immigrants were significantly less likely to have health problems than their US-born counterparts. In other words, US-born respondents were overall more likely to have any health condition, multimorbidity, and a higher incidence rate of conditions. However, we examined a total of 68 conditions, and found that the paradox only applied to specific conditions, which were depression, nerve problem causing numbness or pain, and obesity. As described in the existing literature, our findings may be explained by the possibility that healthy individuals are more capable of migrating to the US (e.g., having greater functioning, possessing the financial means and social resources) than individuals with chronic health conditions [8]. It is also possible that unhealthy migrants residing in the US return to their countries of origin after getting sick, resulting in “salmon bias,” though evidence for this hypothesis is modest, mixed, or lacking [9]. Another possibility is that immigrants may retain aspects of their culture that prove to be salutary, protecting against low socioeconomic status and other health risk factors in the US; studies have shown a correlation between acculturation and poor health (including body mass index, self-rated health, oral health) [6,10,11]. The aforementioned hypotheses are not mutually exclusive, and a combination of factors likely shape the immigrant paradox.

Our findings should be interpreted in light of their limitations. First, the data were cross-sectional, and did not allow us to ascertain the order of events or make causal inferences. The cross-sectional data do not capture aspects of immigration (such as acculturation) and health trajectories, which can change over time. In this study, we did not have access to data on the duration of residence in the US, which is a critical factor given that the health advantage seems to wane as immigrants become more acculturated and adopt the health behaviors of the host country. 

Second, all health conditions were self-reported, and may have been subject to biases. Respondents may not have remembered their diagnoses (i.e., recall bias) or may have been reluctant to disclose conditions such as infectious diseases or psychiatric disorders (i.e., social desirability bias). Further, it is possible that respondents may have had conditions that they were unaware of due to underutilization of formal treatment. Studies have shown that immigrants may underutilize mental health services, due to the cost of care, language barriers, lack of access to health insurance, and stigma [12], and my not receive formal diagnoses or become aware of their health problems.

Third, we lacked information about the context and experience of immigration, which can modify the relationship between immigrant status and health. The association may be conditional on years in the country, age of immigration (childhood vs. adulthood), where immigrants came from, under what conditions (e.g., refugees, skilled professionals), where they geographically settled, and how they were received (i.e., context of reception) [13]. As of 2018, around a quarter of immigrants came from Mexico [1]; however, Asian immigrants are expected to surpass Hispanic immigrants by 2055, and these changing migration patterns may reveal a new portrait of immigrant health in the years to come.

Finally, the ALP was representative of the general US adult population, but may have systematically excluded individuals who were homeless, undocumented, or reluctant/unable to participate in online surveys. Along these lines, the survey was only administered in English, and therefore excluded a substantial portion of the immigrant population who have limited English proficiency. Undocumented immigrants make up almost a quarter of the immigrant population in the U.S., and anti-immigration policies over the past twenty years may be linked to a decline in the access to healthcare for this subpopulation, and may have had detrimental effects on the mental health outcomes, including anxiety, depression, and post-traumatic stress disorder [14]. It is unknown the extent to which undocumented immigrants were included in the ALP sample.

## 5. Conclusions

The findings from this 2019 survey show that immigrants tended to be healthier than their US-born counterparts overall, but specifically with respect to nerve problem causing numbness or pain, depression, and obesity. These findings comport with prior literature that shows a health advantage among immigrants, and reinforces the immigrant paradox, though more longitudinal studies are needed comparing individuals who migrate to those who remain in the country of origin to ascertain the health impact of immigration. Still, it is important to acknowledge that immigrants (especially undocumented) tend to make larger out-of-pocket payments and smaller insurance-sourced payments for healthcare per capita when compared to nonimmigrants [15]. Thus, healthcare systems should be wary of interpreting a health advantage as an absence of need.

## Figures and Tables

**Table 1:** Descriptive statistics

	US-Born (*n* = 2328)	Foreign-Born (*n* = 227)	Total (*n* = 2555)	*p*-Value
**Health Conditions**				
At least one health condition	1657 (71.9%)	137 (60.4%)	1794 (70.9%)	0.00
Two or more health conditions	1225 (53.1%)	97 (42.7%)	1322 (52.2%)	0.00
Count	2.54 (2.40–2.67)	1.94 (1.62–2.26)	2.49 (1.25–2.62)	0.00
**Sociodemographic Characteristics**				
Age				
18–25	12 (0.52%)	5 (2.2%)	17 (0.67%)	0.00
26–44	459 (19.72%)	69 (30.4%)	528 (20.67%)	
45–64	1055 (45.32%)	101 (44.49%)	1156 (45.24%)	
65+	802 (34.45%)	52 (22.91%)	854 (33.42%)	
Sex				
Male	994 (42.9%)	108 (47.6%)	1102 (43.3%)	0.22
Female	1334 (57.30%)	119 (52.42%)	1453 (56.87%)	
Race				
White	1738 (74.66%)	70 (30.84%)	1808 (70.76%)	0.00
Latinx	292 (12.54%)	81 (35.68%)	373 (14.6%)	
Black	226 (9.71%)	22 (9.69%)	248 (9.71%)	
Other	72 (3.09%)	54 (23.79%)	126 (4.93%)	
Household Income				
<$25,000	355 (15.28%)	45 (19.82%)	400 (15.68%)	0.57
$25,000–49,999	540 (23.24%)	50 (22.03%)	590 (23.13%)	
$50,0000–74,999	504 (21.69%)	48 (21.15%)	552 (21.64%)	
$75,000–99,999	266 (11.45%)	20 (8.81%)	286 (11.21%)	
$100,000–124,999	256 (11.02%)	20 (8.81%)	276 (10.82%)	
$125,000–199,999	273 (11.75%)	29 (12.78%)	302 (11.84%)	
$200,000+	130 (5.59%)	15 (6.61%)	145 (5.68%)	
Education				
Less than high school	9 (0.39%)	3 (1.32%)	12 (0.47%)	0.03
Some high school but no diploma	49 (2.1%)	6 (2.64%)	55 (2.15%)	
High school graduate or equivalent	275 (11.81%)	16 (7.05%)	291 (11.39%)	
Some college but no degree	515 (22.12%)	40 (17.62%)	555 (21.72%)	
Professional school degree	81 (3.48%)	11 (4.85%)	92 (3.6%)	
Associate’s degree	311 (13.36%)	30 (13.22%)	341 (13.35%)	
Bachelor’s degree	610 (26.2%)	76 (33.48%)	686 (26.85%)	
Master’s degree	409 (17.57%)	34 (14.98%)	443 (17.34%)	
Doctoral degree	69 (2.96%)	11 (4.85%)	80 (3.13%)	
Insurance				
No	138 (6.0%)	23 (10.13%)	161 (6.35%)	0.02
Yes	2171 (94.02%)	204 (89.87%)	2375 (93.65)	

## Data Availability

All data used in this study are available at: https://www.rand.org/research/data/alp.html.

## Supplementary Materials

The following are available online at https://www.mdpi.com/article/10.3390/ijerph18094619/s1, Table S1: Descriptive Statistics, Table S2: Bivariate models, Table S3: Adjusted models.
