# Supplementary material for: Revisiting the Immigrant Epidemiological Paradox: Findings from the American Panel of Life 2019
Source: Int J Environ Res Public Health. 2021 Apr 27;18(9):4619. doi: 10.3390/ijerph18094619 (PMC8123790; doi:10.3390/ijerph18094619)
Supplement: Supplementary file 1 [file ijerph-18-04619-s001.zip › ijerph-1171667-SI.pdf]

Table S1. Descriptive statistics.

Specific Health conditions	US Born (n = 2328)	Foreign Born (n = 227)	Total (N = 2555)	p-Value
Arthritis	735 (33.1%)	47 (21.7%)	782 (32.1%)	**0.00**
Back pain due to spinal stenosis	158 (7.1%)	9 (4.1%)	167 (6.9%)	0.10
Back pain due to other causes	611 (27.5%)	52 (24.0%)	663 (27.2%)	0.26
Neck pain	373 (16.8%)	26 (12.0%)	399 (16.4%)	0.07
Fibromyalgia	75 (3.4%)	8 (3.7%)	83 (3.4%)	0.81
Lupus	19 (0.9%)	1 (0.5%)	20 (0.8%)	0.54
Ehlers-Danlos Syndrome	2 (0.1%)	0 (0.0%)	2 (0.1%)	0.66
Deformity of limb	7 (0.3%)	1 (0.5%)	8 (0.3%)	0.72
Amputation of limb	12 (0.5%)	1 (0.5%)	13 (0.5%)	0.88
Severe burn	4 (0.2%)	0 (0.0%)	4 (0.2%)	0.53
Other muscle or connective tissue disorder	87 (3.9%)	6 (2.8%)	93 (3.8%)	0.40
Other bone or joint disorder	167 (7.5%)	11 (5.1%)	178 (7.3%)	0.18
Other injury	131 (5.9%)	8 (3.7%)	139 (5.7%)	0.18
Multiple sclerosis	17 (0.8%)	1 (0.5%)	18 (0.7%)	0.62
Seizure disorder	10 (0.5%)	0 (0.0%)	10 (0.4%)	0.32
Parkinson’s Disease	7 (0.3%)	0 (0.0%)	7 (0.3%)	0.41
Stroke (or effects of a prior stroke)	32 (1.4%)	4 (1.8%)	36 (1.5%)	0.64
Migraine	224 (10.1%)	21 (9.7%)	245 (10.1%)	0.84
Blindness	13 (0.6%)	0 (0.0%)	13 (0.5%)	0.26
Deafness	44 (2.0%)	5 (2.3%)	49 (2.0%)	0.75
**Nerve problem causing numbness or pain**	**330 (14.9%)**	**19 (8.8%)**	**349 (14.3%)**	**0.01**
Other nervous system disorder	52 (2.3%)	6 (2.8%)	58 (2.4%)	0.70
Heart failure	46 (2.1%)	2 (0.9%)	48 (2.0%)	0.24
Coronary artery disease	77 (3.5%)	4 (1.8%)	81 (3.3%)	0.20
Heart valve dysfunction	31 (1.4%)	4 (1.8%)	35 (1.4%)	0.60
Peripheral arterial disease	37 (1.7%)	5 (2.3%)	42 (1.7%)	0.49
Abnormal heart rhythm	32 (1.4%)	2 (0.9%)	34 (1.4%)	0.53
Lymphedema	21 (0.9%)	3 (1.4%)	24 (1.0%)	0.54
**Other heart or circulatory system disorder**	**147 (6.6%)**	**5 (2.3%)**	**152 (6.2%)**	**0.01**
Asthma	227 (10.2%)	14 (6.5%)	241 (9.9%)	0.07
Chronic obstructive pulmonary disease/emphysema	72 (3.2%)	4 (1.8%)	76 (3.1%)	0.26
Interstitial lung disease/pulmonary fibrosis	2 (0.1%)	0 (0.0%)	2 (0.1%)	0.66
Pulmonary hypertension	32 (1.4%)	1 (0.5%)	33 (1.4%)	0.23
Other lung disease	31 (1.4%)	2 (0.9%)	33 (1.4%)	0.56
Chron’s disease	13 (0.6%)	2 (0.9%)	15 (0.6%)	0.55
Ulcerative Colitis	24 (1.1%)	0 (0.0%)	24 (1.0%)	0.12
Liver disease/Cirrhosis	14 (0.6%)	0 (0.0%)	14 (0.6%)	0.24
Chronic kidney disease (not on dialysis)	46 (2.1%)	2 (0.9%)	48 (2.0%)	0.24
Chronic kidney disease (on dialysis)	5 (0.2%)	2 (0.9%)	7 (0.3%)	0.07
**Other gastrointestinal disorder**	**233 (10.5%)**	**12 (5.5%)**	**245 (10.1%)**	**0.02**
Other kidney or bladder disorder	70 (3.2%)	5 (2.3%)	75 (3.1%)	0.49
HIV	18 (0.8%)	1 (0.5%)	19 (0.8%)	0.58
Hepatitis	13 (0.6%)	0 (0.0%)	13 (0.5%)	0.26
Other infectious diseases	18 (0.8%)	3 (1.4%)	21 (0.9%)	0.39
Schizophrenia	7 (0.3%)	2 (0.9%)	9 (0.4%)	0.16
Bipolar disorder	53 (2.4%)	3 (1.4%)	56 (2.3%)	0.34
**Depression**	**408 (18.4%)**	**24 (11.1%)**	**432 (17.7%)**	**0.01**
Anxiety	412 (18.6%)	30 (13.8%)	442 (18.2%)	0.08

Attention Deficit/Hyperactivity Disorder	63 (2.8%)	8 (3.7%)	71 (2.9%)	0.48
Post-traumatic stress disorder	106 (4.8%)	10 (4.6%)	116 (4.8%)	0.91
Personality disorder	5 (0.2%)	0 (0.0%)	5 (0.2%)	0.48
Alzheimer's disease	1 (0.0%)	0 (0.0%)	1 (0.0%)	0.75
Other dementia	12 (0.5%)	1 (0.5%)	13 (0.5%)	0.88
Other mental or cognitive disorder	27 (1.2%)	0 (0.0%)	27 (1.1%)	0.10
Alcohol dependence	29 (1.3%)	3 (1.4%)	32 (1.3%)	0.93
Opioid dependence	13 (0.6%)	0 (0.0%)	13 (0.5%)	0.26
Other Substance Use Disorder	13 (0.6%)	0 (0.0%)	13 (0.5%)	0.26
Cancer	92 (4.1%)	9 (4.1%)	101 (4.1%)	1.00
Diabetes	298 (13.4%)	35 (16.1%)	333 (13.7%)	0.27
**Obesity**	**406 (18.3%)**	**25 (11.5%)**	**431 (17.7%)**	**0.01**
Cerebral Palsy	2 (0.1%)	1 (0.5%)	3 (0.1%)	0.14
Sleep disorder	257 (11.6%)	24 (11.1%)	281 (11.5%)	0.82
Chronic fatigue syndrome	45 (2.0%)	3 (1.4%)	48 (2.0%)	0.51
Chronic pain	177 (8.0%)	12 (5.5%)	189 (7.8%)	0.20
Sickle Cell Anemia	4 (0.2%)	1 (0.5%)	5 (0.2%)	0.38
Immune Deficiency	22 (1.0%)	2 (0.9%)	24 (1.0%)	0.92
Other blood disorder	40 (1.8%)	7 (3.2%)	47 (1.9%)	0.15
Other health problem	153 (6.9%)	16 (7.4%)	169 (6.9%)	0.79

p-values reflect chi2 test for binary/categorical variables.

Table S2. Bivariate models.

Table : Arthritis

	OR [95% CI]	P-Value
**Immigrant status**		
Foreign born	**0.56 [0.41–0.77]**	**0.00**
US born	1.00	

Table : Back pain due to spinal stenosis

	OR [95% CI]	P-Value
**Immigrant status**		
Foreign born	0.56 [0.28–1.12]	0.10
US born	1.00	

Table : Back pain due to other causes

	OR [95% CI]	P-Value
**Immigrant Status**		
Foreign born	0.83 [0.61–1.14]	0.24
US born	1.00	

Table : Neck pain

	OR [95% CI]	P-Value
**Immigrant Status**		
Foreign born	0.67 [0.45–1.01]	0.06
US born	1.00	

Table : Fibromyalgia

	OR [95% CI]	P-Value
**Immigrant Status**		
Foreign born	1.09 [0.48–2.49]	0.83
US born	1.00	

Table : Lupus

	OR [95% CI]	P-Value
**Immigrant Status**		
Foreign born	0.54 [0.07–4.08]	0.55
US born	1.00	

Table : Ehlers-Danlos Syndrome

	OR [95% CI]	P-Value
**Immigrant Status**		
Foreign born	1.00 [0.00–0.00]	.
US born	1.00	

Table : Deformity of limb

	OR [95% CI]	P-Value
**Immigrant Status**		
Foreign born	1.46 [0.18–12.18]	0.72
US born	1.00	

Table : Amputation of limb

	OR [95% CI]	P-Value
**Immigrant Status**		
Foreign born	0.85 [0.11–6.79]	0.88
US born	1.00	

Table : Severe burn

	OR [95% CI]	P-Value
**Immigrant Status**		
Foreign born	1.00 [0.00–0.00]	.
US born	1.00	

Table : Other muscle or connective tissue disorder

	OR [95% CI]	P-Value
**Immigrant Status**		
Foreign born	0.70 [0.30–1.61]	0.40
US born	1.00	

Table : Other bone or joint disorder

	OR [95% CI]	P-Value
**Immigrant Status**		
Foreign born	0.66 [0.34–1.27]	0.21
US born	1.00	

Table : Other injury

	OR [95% CI]	P-Value
**Immigrant Status**		
Foreign born	0.61 [0.29–1.27]	0.18
US born	1.00	

Table : Multiple sclerosis

	OR [95% CI]	P-Value
**Immigrant Status**		
Foreign born	0.60 [0.08–4.63]	0.62
US born	1.00	

Table : Seizure disorder

	OR [95% CI]	P-Value
**Immigrant Status**		
Foreign born	1.00 [0.00–0.00]	.
US born	1.00	

Table : Parkinson's Disease

	OR [95% CI]	P-Value
**Immigrant Status**		
Foreign born	1.00 [0.00–0.00]	.
US born	1.00	

Table : Stroke (or effects of a prior stroke)

	OR [95% CI]	P-Value
**Immigrant Status**		
Foreign born	1.28 [0.44–3.71]	0.65
US born	1.00	

Table : Migraine

	OR [95% CI]	P-Value
**Immigrant Status**		
Foreign born	0.95 [0.60–1.51]	0.84
US born	1.00	

Table : Blindness

	OR [95% CI]	P-Value
**Immigrant Status**		
Foreign born	1.00 [0.00–0.00]	.
US born	1.00	

Table : Deafness

	OR [95% CI]	P-Value
**Immigrant Status**		
Foreign born	1.17 [0.45–3.04]	0.75
US born	1.00	

Table : Nerve problem causing numbness or pain

	OR [95% CI]	P-Value
**Immigrant Status**		
Foreign born	**0.55 [0.34–0.89]**	**0.02**
US born	1.00	

Table : Other nervous system disorder

	OR [95% CI]	P-Value
**Immigrant Status**		
Foreign born	1.18 [0.51–2.77]	0.70

US born	1.00	

Table : Heart failure

	OR [95% CI]	P-Value
**Immigrant Status**		
Foreign born	0.44 [0.10–1.85]	0.26
US born	1.00	

Table : Coronary artery disease

	OR [95% CI]	P-Value
**Immigrant Status**		
Foreign born	0.52 [0.18–1.48]	0.22
US born	1.00	

Table : Heart valve dysfunction

	OR [95% CI]	P-Value
**Immigrant Status**		
Foreign born	1.32 [0.45–3.92]	0.61
US born	1.00	

Table : Peripheral arterial disease

	OR [95% CI]	P-Value
**Immigrant Status**		
Foreign born	1.39 [0.53–3.63]	0.50
US born	1.00	

Table : Abnormal heart rhythm

	OR [95% CI]	P-Value
**Immigrant Status**		
Foreign born	0.64 [0.15–2.72]	0.54
US born	1.00	

Table : Lymphedema

	OR [95% CI]	P-Value
**Immigrant Status**		
Foreign born	1.47 [0.44–4.93]	0.54
US born	1.00	

Table : Other heart or circulatory system disorder

	OR [95% CI]	P-Value
**Immigrant Status**		
Foreign born	**0.33 [0.14–0.79]**	**0.01**
US born	1.00	

Table : Asthma

	OR [95% CI]	P-Value
**Immigrant Status**		
Foreign born	0.60 [0.34–1.08]	0.09
US born	1.00	

Table : Chronic obstructive pulmonary disease/emphysema

	OR [95% CI]	P-Value
**Immigrant Status**		
Foreign born	0.56 [0.21–1.48]	0.24
US born	1.00	

Table : Interstitial lung disease/pulmonary fibrosis

	OR [95% CI]	P-Value
**Immigrant Status**		
Foreign born	1.00 [0.00–0.00]	.
US born	1.00	

Table : Pulmonary hypertension

	OR [95% CI]	P-Value
**Immigrant Status**		
Foreign born	0.32 [0.04–2.35]	0.26
US born	1.00	

Table : Other lung disease

	OR [95% CI]	P-Value
**Immigrant Status**		
Foreign born	0.66 [0.17–2.57]	0.55
US born	1.00	

Table : Chron's disease

	OR [95% CI]	P-Value
**Immigrant Status**		
Foreign born	1.58 [0.21–12.07]	0.66
US born	1.00	

Table : Ulcerative Colitis

	OR [95% CI]	P-Value
**Immigrant Status**		
Foreign born	1.00 [0.00–0.00]	.
US born	1.00	

Table : Liver disease/Cirrhosis

	OR [95% CI]	P-Value
**Immigrant Status**		
Foreign born	1.00 [0.00–0.00]	.
US born	1.00	

Table : Chronic kidney disease (not on dialysis)

	OR [95% CI]	P-Value
**Immigrant Status**		
Foreign born	0.44 [0.10–1.85]	0.26
US born	1.00	

Table : Chronic kidney disease (on dialysis)

	OR [95% CI]	P-Value
**Immigrant Status**		

Foreign born	4.12 [0.79–21.50]	0.09
US born	1.00	

Table : Other gastrointestinal disorder

	OR [95% CI]	P-Value
**Immigrant Status**		
Foreign born	**0.50 [0.27–0.91]**	**0.02**
US born	1.00	

Table : Other kidney or bladder disorder

	OR [95% CI]	P-Value
**Immigrant Status**		
Foreign born	0.72 [0.29–1.83]	0.50
US born	1.00	

Table : HIV

	OR [95% CI]	P-Value
**Immigrant Status**		
Foreign born	0.57 [0.07–4.34]	0.58
US born	1.00	

Table : Hepatitis

	OR [95% CI]	P-Value
**Immigrant Status**		
Foreign born	1.00 [0.00–0.00]	.
US born	1.00	

Table : Other infectious diseases

	OR [95% CI]	P-Value
**Immigrant Status**		
Foreign born	1.71 [0.49–6.00]	0.40
US born	1.00	

Table : Any infectious disease

	OR [95% CI]	P-Value
**Immigrant Status**		
Foreign born	0.91 [0.51–1.61]	0.74
US born	1.00	

Table : Schizophrenia

	OR [95% CI]	P-Value
**Immigrant Status**		
Foreign born	2.94 [0.60–14.28]	0.18
US born	1.00	

Table : Bipolar disorder

	OR [95% CI]	P-Value
**Immigrant Status**		
Foreign born	0.57 [0.18–1.86]	0.35
US born	1.00	

Table : Depression

	OR [95% CI]	P-Value
**Immigrant Status**		
Foreign born	**0.55 [0.36–0.84]**	**0.01**
US born	1.00	

Table : Anxiety

	OR [95% CI]	P-Value
**Immigrant Status**		
Foreign born	0.70 [0.47–1.06]	0.09
US born	1.00	

Table : Attention Deficit/Hyperactivity Disorder

	OR [95% CI]	P-Value
**Immigrant Status**		
Foreign born	1.31 [0.64–2.69]	0.46
US born	1.00	

Table : Post-traumatic stress disorder

	OR [95% CI]	P-Value
**Immigrant Status**		
Foreign born	0.96 [0.50–1.86]	0.91
US born	1.00	

Table : Personality disorder

	OR [95% CI]	P-Value
**Immigrant Status**		
Foreign born	1.00 [0.00–0.00]	.
US born	1.00	

Table : Alzheimer's disease

	OR [95% CI]	P-Value
**Immigrant Status**		
Foreign born	1.00 [0.00–0.00]	.
US born	1.00	

Table : Other dementia

	OR [95% CI]	P-Value
**Immigrant Status**		
Foreign born	0.85 [0.11–6.78]	0.88
US born	1.00	

Table : Other mental or cognitive disorder

	OR [95% CI]	P-Value
**Immigrant Status**		
Foreign born	1.00 [0.00–0.00]	.
US born	1.00	

Table : Alcohol dependence

	OR [95% CI]	P-Value

**Immigrant Status**		
Foreign born	1.06 [0.33–3.43]	0.92
US born	1.00	

Table : Opioid dependence

	OR [95% CI]	P-Value
**Immigrant Status**		
Foreign born	1.00 [0.00–0.00]	.
US born	1.00	

Table : Other Substance Use Disorder

	OR [95% CI]	P-Value
**Immigrant Status**		
Foreign born	1.00 [0.00–0.00]	.
US born	1.00	

Table : Cancer

	OR [95% CI]	P-Value
**Immigrant Status**		
Foreign born	1.00 [0.48–2.09]	1.00
US born	1.00	

Table : Diabetes

	OR [95% CI]	P-Value
**Immigrant Status**		
Foreign born	1.24 [0.83–1.86]	0.30
US born	1.00	

Table : Obesity

	OR [95% CI]	P-Value
**Immigrant Status**		
Foreign born	**0.58 [0.39–0.87]**	**0.01**
US born	1.00	

Table : Cerebral Palsy

	OR [95% CI]	P-Value
**Immigrant Status**		
Foreign born	5.13 [0.45–57.92]	0.19
US born	1.00	

Table : Sleep disorder

	OR [95% CI]	P-Value
**Immigrant Status**		
Foreign born	0.95 [0.62–1.46]	0.81
US born	1.00	

Table : Chronic fatigue syndrome

	OR [95% CI]	P-Value
**Immigrant Status**		
Foreign born	0.68 [0.21–2.22]	0.52
US born	1.00	

Table : Chronic pain

	OR [95% CI]	P-Value
**Immigrant Status**		
Foreign born	0.67 [0.37–1.23]	0.20
US born	1.00	

Table : Sickle Cell Anemia

	OR [95% CI]	P-Value
**Immigrant Status**		
Foreign born	2.56 [0.28–23.14]	0.40
US born	1.00	

Table : Immune Deficiency

	OR [95% CI]	P-Value
**Immigrant Status**		
Foreign born	0.93 [0.21–4.11]	0.92
US born	1.00	

Table : Other blood disorder

	OR [95% CI]	P-Value
**Immigrant Status**		
Foreign born	1.82 [0.79–4.18]	0.16
US born	1.00	

Table : Other health problem

	OR [95% CI]	P-Value
**Immigrant Status**		
Foreign born	1.07 [0.62–1.85]	0.80
US born	1.00	

Table S3. Adjusted models.

Table : Arthritis

Variable	OR [95% CI]	P-Value
Immigrant Status		
Foreign Born	0.71 [0.50–1.01]	0.06
US Born	1.00	

Table : Nerve problem causing numbness or pain

Variable	OR [95% CI]	P-Value
Immigrant Status		
**Foreign Born**	**0.55 [0.33–0.93]**	**0.02**
US Born	1.00	

Table : Other heart or circulatory system disorder

Variable	OR [95% CI]	P-Value
Immigrant Status		
Foreign Born	0.44 [0.17–1.16]	0.10
US Born	1.00	

Table : Depression

Variable	OR [95% CI]	P-Value
Immigrant Status		
**Foreign Born**	**0.50 [0.32–0.80]**	**0.00**
US Born	1.00	

Table : Obesity

Variable	OR [95% CI]	P-Value
Immigrant Status		
**Foreign Born**	**0.63 [0.40–0.98]**	**0.04**
US Born	1.00	
